# Supplementary material for: Extracellular HSP90α Induces MyD88-IRAK Complex-Associated IKKα/β−NF-κB/IRF3 and JAK2/TYK2−STAT-3 Signaling in Macrophages for Tumor-Promoting M2-Polarization
Source: Cells. 2022 Jan 11;11(2):229. doi: 10.3390/cells11020229 (PMC8774043; doi:10.3390/cells11020229)
Supplement: Supplementary file 1 [file cells-11-00229-s001.zip › cells-1505413-supplementary.pdf]

Supplementary Materials:

Table S1. The primers and PCR conditions for RT-PCR. All reactions started at 95°C for 5 min and terminated at 72°C for 7 min.

Gene	Primer Sequence	PCR Condition & Product Size
TNF-α (human)	Forward: 5’-CCC-AGG-CAG-TCA-GAT-CAT-CTT-3’<br>Reverse: 5’-TCT-CAG-CTC-CAC-GCC-ATT-3’	95°C (30 sec), 62°C (40 sec), and 72°C (40 sec) for 26 cycles; 140 bp
TNF-α (mouse)	Forward: 5’-GAG-GCA-ACC-TGA-CCA-CTC-TC-3’<br>Reverse: 5’-AGC-CCC-CAG-TCT-GAT-TCC-TT-3’	95°C (30 sec), 62°C (45 sec), and 72°C (40 sec) for 33 cycles; 212 bp
IL-1β (human)	Forward: 5’-ATT-CTG-ATG-AGC-AAC-CGC-TT-3’<br>Reverse: 5’-GCA-CAC-CAG-TCC-AAA-TTG-AA-3’	95°C (30 sec), 52°C (40 sec), and 72°C (40 sec) for 30 cycles; 156 bp
IL-1β (mouse)	Forward: 5’-CAG-GCA-GGC-AGT-ATC-ACT-CA-3’<br>Reverse: 5’-AGC-TCA-TAT-GGG-TCC-GAC-AG-3’	95°C (30 sec), 59°C (40 sec), and 72°C (40 sec) for 32 cycles; 230 bp
CD163 (human)	Forward: 5’-GAA-TAT-CAA-AAT-TGC-AAT-CAT-AGG-G-3’<br>Reverse: 5’-GTT-CAT-TTG-CTT-TGC-TTT-AGT-AAG-C-3’	95°C (30 sec), 62°C (40 sec), and 72°C (40 sec) for 32 cycles; 265 bp
CD163 (mouse)	Forward: 5’-CAT-GTC-TCT-GAG-GCT-GAC-CA-3’<br>Reverse: 5’-TGC-ACA-CGA-TCT-ACC-CAC-AT-3’	95°C (30 sec), 59°C (40 sec), and 72°C (40 sec) for 38 cycles; 219 bp
CD204 (human)	Forward: 5’-ATA-GCT-TCC-AGA-TTA-CAA-AGG-CC-3’<br>Reverse: 5’-AGA-AAA-ATA-AGC-TCC-CTG-GTC-C-3’	95°C (30 sec), 56°C (40 sec), and 72°C (40 sec) for 32 cycles; 328 bp
CD204 (mouse)	Forward: 5’-CTG-GAC-AAA-CTG-GTC-CAC-CT-3’<br>Reverse: 5’-CCT-AGA-CTC-CGG-CAG-ACA-AC-3’	95°C (30 sec), 60°C (45 sec), and 72°C (40 sec) for 38 cycles; 168 bp
IL-10 (human)	Forward: 5’-ATG-CCC-CAA-GCT-GAG-AAC-CAA-GAC-CCA-3’<br>Reverse: 5’-TCT-CAA-GGG-GCT-GGG-TCA-GCT-ATC-CCA-3’	95°C (30 sec), 60°C (40 sec), and 72°C (40 sec) for 30 cycles; 365 bp
IL-10 (mouse)	Forward: 5’-CCA-AGC-CTT-ATC-GGA-AAT-GA-3’<br>Reverse: 5’-TTT-TCA-CAG-GGG-AGA-AAT-CG-3’	95°C (30 sec), 62°C (45 sec), and 72°C (40 sec) for 30 cycles; 162 bp
TGF-β (human)	Forward: 5’-CTA-CTA-CGC-CAA-GGA-GGT-CAC-3’<br>Reverse: 5’-TTG-CTG-AGG-TAT-CGC-CAG-GAA-3’	95°C (30 sec), 60°C (40 sec), and 72°C (40 sec) for 30 cycles; 249 bp
TGF-β (mouse)	Forward: 5’-CTG-CTG-CTT-TCT-CCC-TCA-AC-3’<br>Reverse: 5’-GAC-TGG-CGA-GCC-TTA-GTT-TG-3’	95°C (30 sec), 58°C (45 sec), and 72°C (40 sec) for 30 cycles; 183 bp
Sec22b (human)	Forward: 5’-TGA-AGC-TGC-CTT-CCC-TAA-GA-3’<br>Reverse: 5’-GAT-ATT-GGC-CAC-CAT-GAT-CC-3’	95°C (30 sec), 62°C (60 sec), and 72°C (30 sec) for 30 cycles; 235 bp
Syntaxin 18 (human)	Forward: 5’-GCT-CTT-CCT-CGA-CTG-GTA-CG-3’<br>Reverse: 5’-CAT-TGG-TGT-GCA-CTG-TTT-CC-3’	95°C (30 sec), 57°C (60 sec), and 72°C (30 sec) for 32 cycles; 250 bp
VEGF (human)	Forward: 5’-CCC-ACT-GAG-GAG-TCC-AAC-AT-3’<br>Reverse: 5’-TTT-CTT-GCG-CTT-TCG-TTT-TT-3’	95°C (30 sec), 56°C (40 sec), and 72°C (40 sec) for 35 cycles; 186 bp
VEGF (mouse)	Forward: 5’-CAG-GCT-GCT-GTA-ACG-ATG-AA-3’<br>Reverse: 5’-TTT-CTT-GCG-CTT-TCG-TTT-TT-3’	95°C (30 sec), 56°C (40 sec), and 72°C (40 sec) for 35 cycles; 221 bp
PDGF (human)	Forward: 5’-ACA-CGA-GCA-GTG-TCA-AGT-GC-3’<br>Reverse: 5’-TCT-GGT-TGG-CTG-CTT-TAG-GT-3’	95°C (30 sec), 56°C (40 sec), and 72°C (40 sec) for 35 cycles; 250 bp
PDGF (mouse)	Forward: 5’-TCT-GCC-TAG-GAC-CTT-CCT-GA-3’<br>Reverse: 5’-TCG-ACG-AAA-ATG-TCA-TCC-AA-3’	95°C (30 sec), 56°C (40 sec), and 72°C (40 sec) for 35 cycles; 171 bp
bFGF (human)	Forward: 5’-TGC-TGG-TGA-TGG-GAG-TTG-TA-3’<br>Reverse: 5’-CTG-AGT-ATT-CGG-CAA-CAG-CA-3’	95°C (30 sec), 56°C (40 sec), and 72°C (40 sec) for 35 cycles; 181 bp
bFGF (mouse)	Forward: 5’-TGG-CAA-AAT-TTC-CAA-AGG-AG-3’<br>Reverse: 5’-TCT-GGA-AGG-AAG-AGG-CTT-GA-3’	95°C (30 sec), 56°C (40 sec), and 72°C (40 sec) for 35 cycles; 227 bp
GAPDH	Forward: 5’-GAA-GGT-GAA-GGT-CGG-AGT-3’<br>Reverse: 5’-GAA-GAT-GGT-GAT-GGG-ATT-TC-3’	95°C (30 sec), 56°C (40 sec), and 72°C (40 sec) for 25 cycles; 220 bp

Table S2. The primers and PCR conditions for ChIP assays. All reactions started at 94°C for 5 min and terminated at 72°C for 7 min.

Gene	Primer Sequence	PCR Condition & Product Size
CD163 gene promoter region containing NF-κB-binding site (-1337 ~ -1327)	Forward: 5'-GAC-ACA-GGA-AGG-GGA-ACA-TC-3'<br>Reverse: 5'-TGT-GCT-GCA-CCC-ATT-AAC-TC-3'	94°C (30 sec), 55°C (30 sec), and 72°C (30 sec) for 35 cycles; 130 bp
CD163 gene promoter region containing IRF3-binding site (-1448 ~ -1436)	Forward: 5'-TGT-TGC-TAA-TTT-TTG-TTT-CAC-CA-3'<br>Reverse: 5'-TGA-GAC-ACA-GCA-TTT-GTC-ATT-TC-3'	94°C (30 sec), 50°C (30 sec), and 72°C (30 sec) for 35 cycles; 190 bp
CD204 gene promoter region containing NF-κB-binding site (-1256 ~ -1247)	Forward: 5'-GCC-TGT-AAT-CCC-AGC-ACT-TT-3'<br>Reverse: 5'-CAT-CAC-ACC-CGG-CTA-ATT-TT-3'	94°C (30 sec), 55°C (30 sec), and 72°C (30 sec) for 35 cycles; 182 bp
CD204 gene promoter region containing IRF3-binding site (-1753 ~ -1745)	Forward: 5'-CAC-ACC-TAG-CCA-AAC-ATG-GA-3'<br>Reverse: 5'-GAT-GGT-CTT-GAT-CCC-CTG-AC-3'	94°C (30 sec), 55°C (30 sec), and 72°C (30 sec) for 35 cycles; 220 bp
IL-10 gene promoter region containing NF-κB-binding site (-1678 ~ -1668)	Forward: 5'-GGG-AAA-CTA-AGG-CCC-AGA-GA-3'<br>Reverse: 5'-CAG-TGA-CGT-GGA-CAA-ATT-GC-3'	94°C (30 sec), 55°C (30 sec), and 72°C (30 sec) for 35 cycles; 125 bp
IL-10 gene promoter region containing IRF3-binding site (-847 ~ -835)	Forward: 5'-CTT-TGG-GAA-GGG-GAA-GTA-GG-3'<br>Reverse: 5'-GAT-GGG-GTG-GAA-GAA-GTT-GA-3'	94°C (30 sec), 55°C (30 sec), and 72°C (30 sec) for 35 cycles; 120 bp
Sec22b gene promoter region containing STAT-3-binding sites (-213 ~ -205 and -137 ~ -128)	Forward: 5'-CCA-TTA-ACT-CCC-ACC-CTC-CT-3'<br>Reverse: 5'-AAG-GTC-TGG-CTG-GGT-TTT-CT-3'	94°C (30 sec), 56°C (60 sec), and 72°C (30 sec) for 40 cycles; 144 bp

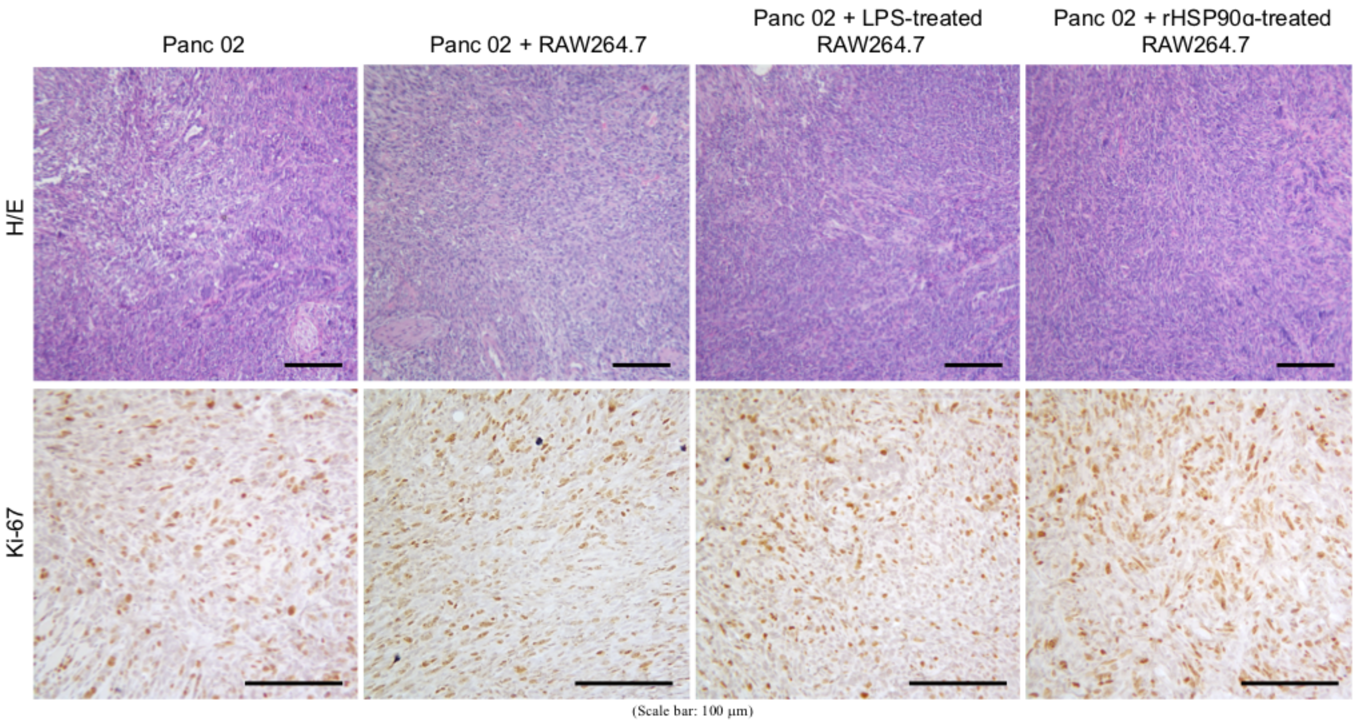

Supplementary Figure S1. IHC of Ki-67 from the tumors formed by Panc 02 cells alone (designated as "Panc 02" group) or Panc 02 cells mixed with PBS-, LPS-, or rHSP90α-treated RAW264.7 cells (designated as "Panc 02 + RAW264.7", "Panc 02 + LPS-treated RAW264.7", and "Panc 02 + rHSP90α-treated RAW264.7" groups, respectively). Despite the tumor growth of "Panc 02" or "Panc 02 + LPS-treated RAW264.7" grafts was much slower than that of "Panc 02 + rHSP90α-treated RAW264.7" grafts, it was noted that abundant Ki-67-positive cells could also be observed from the small tumor tissues of "Panc 02" and "Panc 02 + LPS-treated RAW264.7" groups. Anti-Ki-67 antibody was purchased from Thermo Fisher Scientific (1:200, cat. #RM-9106-S). H/E, Hematoxylin and Eosin staining.
